# Evaluating a Dual Digital Cognitive Behavioral Therapy and Health and Wellness Coaching Intervention for Anxiety and Depression: Single-Arm Pilot Study

**DOI:** 10.2196/92448

**Published:** 2026-06-25

**Authors:** Alex Kirk, Christopher S King, Robert Gallop, Brian D Doan, Lorelei Simpson Rowe, Laura E Randa

**Affiliations:** 1 Toivoa, Inc, 591 Collaboration Way, Newark, DE, 19713, United States, 1 3032108106; 2 Department of Mathematics, West Chester University, West Chester, PA, United States; 3 Department of Psychiatry and Psychology, Mayo Clinic, Rochester, MN, United States

**Keywords:** digital therapeutic, digital cognitive behavioral therapy, virtual mental health, health and wellness coaching, anxiety, depression, disability

## Abstract

**Background:**

Anxiety and depressive disorders remain highly prevalent and insufficiently treated, with many individuals experiencing persistent or untreated symptoms, limited access to evidence-based care, or insufficient support between clinical encounters. Adults with disabilities represent a particularly underserved subpopulation, often facing compounded barriers to mental health care and higher rates of anxiety and depression. Digital therapeutics offer a scalable opportunity to address these gaps by extending structured, evidence-based interventions beyond traditional care settings.

**Objective:**

This pilot study evaluated Rauha, a novel digital therapeutic created by Toivoa Inc, that integrates cognitive behavioral therapy (CBT)–based modules with live weekly sessions led by a National Board-Certified Health and Wellness Coach (NBC-HWC), delivering structured, smartphone-based psychoeducation and interactive therapeutic exercises combined with personalized mental health coaching to support behavior change.

**Methods:**

Thirteen adults with mobility and/or hearing disabilities and clinically elevated anxiety and/or depression were enrolled in a single-arm, within-participants design. Participants completed 8 weeks of CBT modules delivered via smartphone, accompanied by synchronous virtual mental health coaching. Anxiety and depression were assessed using the Hamilton Anxiety and Hamilton Depression Rating Scales, respectively, at baseline, post treatment, and at the 4-week follow-up.

**Results:**

Mean reductions were significant for both anxiety (−13.05, SD 2.51; *P*<.001) and depression (−12.83, SD 1.55; *P*<.001), exceeding thresholds for clinical significance and sustained through follow-up. Post treatment, 84.6% (11/13) of participants showed clinically significant improvement in both anxiety and depression. At follow-up, 76.9% (10/13) and 92.3% (12/13) of participants showed clinically significant improvement in anxiety and depression, respectively. Between baseline and follow-up time points, these reductions corresponded to mean shifts from moderate to mild anxiety on the Hamilton Anxiety Rating Scale and from moderate to mild/nondepressed on the Hamilton Depression Rating Scale. Participants reported strongly favorable acceptability, experience, and usability ratings for the Rauha treatment program, demonstrating 100% treatment retention and an average replay rate of 5.5 for personalized smartphone content.

**Conclusions:**

The findings suggest that a combined digital CBT and NBC-HWC approach can yield clinically meaningful and durable symptom reductions in depression and anxiety, coupled with high user acceptability and engagement, for adults with disabilities. These findings provide preliminary evidence supporting Rauha as a scalable, evidence-informed mental health intervention with the strong potential to improve access and address key barriers to care.

## Introduction

### Digital Interventions for Adults with Disabilities

About 18.2% and 21.4% of adults reported symptoms of anxiety and depression, respectively [1]. These conditions are often undertreated, partly due to many obstacles prohibiting treatment initiation and engagement, resulting in a substantial unmet need for effective and accessible mental health interventions [2]. Among underserved populations are adults with physical disabilities or functional limitations, who comprise a large proportion of the US population, with over 40% of those aged 45 to 64 years reporting such limitations, a 21% increase in prevalence from 2002 to 2016 [3]. This growing population faces compounded barriers to care, including limited access to evidence-based therapies, highlighting the critical need for scalable interventions tailored to both mental health and functional challenges. Additionally, this population is at higher risk of mental health difficulties than those without a disability, with recent data showing that 32.9% of those with disabilities report significant mental distress as compared with 7.2% of those without disabilities [4].

Unfortunately, individuals with disabilities and functional impairments also face significant barriers in accessing adequate treatment, including limited treatment options, a lack of adequate training among providers concerning the unique needs of those with disabilities, and limited transportation capabilities [5,6]. Further worsening this public health problem is a severe shortage of mental health providers. On average, within the United States, only 26.4% of all mental health care needs are being met based on provider volume and availability [7]. These provider shortages strongly contribute to the 2023 finding that 46% of US adults with a mental disorder did not receive care for their condition [8].

Fortunately, evidence suggests that these obstacles are surmountable. For example, research has shown that cognitive behavioral therapy (CBT) and mindfulness-based therapies are strong approaches for this population [9-11]. Furthermore, virtual mental health interventions for those with disabilities can address obstacles related to mobility or transportation [12,13], reduce wait times, and increase overall access [14-16]. Self-guided CBT has shown significant promise in treating adults with mild-to-severe mental health symptoms without requiring a licensed provider [17,18].

In response to the rapid expansion of digital health technologies, the digital therapeutics (DTx) category has emerged as a distinct class of evidence-based interventions. Although definitions vary, DTx is largely characterized as a digital, evidence-based medical intervention delivered directly to patients to prevent, treat, or manage a disease or disorder using validated clinical frameworks [19]. As it relates to mental health, DTx and closely related evidence-based digital interventions demonstrate significant promise in reducing symptom severity across multiple conditions [20-24]. However, there are notable limitations, including high rates of attrition [25], often attributed to symptom severity beyond the scope of self-guided DTx capabilities, technological limitations, and insufficient personalization or support [26,27]. Importantly, evidence suggests that attrition can be reduced and adherence improved through the inclusion of human support elements that enhance accountability, personalization, and timely response to changes in treatment needs [28,29]. However, despite their promise, many existing digital platforms remain inadequately accessible for individuals with disabilities, inadvertently reproducing the very barriers they aim to reduce [26,30]. As such, designing DTx in alignment with established accessibility standards, such as the Web Content Accessibility Guidelines (WCAG) 2.2 [31], is therefore not a peripheral design consideration but a foundational requirement for equitable delivery of evidence-based care.

Beyond digital solutions, another step in expanding mental health care has been the significant increase in health and wellness coaches, such as National Board-Certified Health and Wellness Coaches (NBC-HWC). Health and wellness coaches are professionals who support patients in making behavioral changes according to self-chosen health goals and needs [32]. In terms of efficacy, a recent meta-analysis supports the use of health and wellness coaches as a stand-alone behavioral intervention in improving mental health outcomes among those with medical and psychological conditions [33]. In addition, adding a health coach to self-guided CBT significantly improves mental health outcomes over and above self-guided CBT alone [34]. At a systemic level, the use of health and wellness coaches is a promising avenue for improving flexible access to mental health care, given that these sessions are significantly shorter than traditional psychotherapy, typically around 30 minutes [32,35], allowing for more patients to receive care per single coach when compared to licensed mental health providers.

In summary, although adults with disabilities face significant mental health concerns and obstacles to accessing adequate care, ongoing innovations in technology, treatment delivery, and types of care show promising results in better reaching and treating this underserved population.

### Development of the Rauha Mental Health Program

Rauha is a smartphone-based DTx developed to deliver flexible, accessible, and evidence-based mental health care for adults with anxiety-related and depression-related symptoms, including those with functional or sensory limitations. The Rauha DTx is a Health Insurance Portability and Accountability Act (HIPAA)–compliant software, certified with WCAG 2.2 AA accessibility standards. This certification ensures usability for individuals with functional, sensory, or other accessibility challenges, as only 5.2% of the top 1 million websites pass WCAG accessibility testing [36]. Per WCAG principles, Rauha is perceivable, operable, understandable, and robust [31]. Therapeutic content is delivered across text, audio, and video, with captions, transcripts, and high-contrast scalable text to support perceivability. The platform allows full keyboard navigation and works with assistive technologies, avoiding reliance on time-sensitive interactions. Content is written in plain language and presented in a consistent, structured format. The system was tested across devices and browsers and designed to work with common accessibility tools.

The clinical program of Rauha consists of 2 integrated components: (1) asynchronous, self-guided, interactive modules and exercises rooted in standard CBT [37], delivered digitally via recorded sessions by a licensed psychologist, allowing users to listen, review, and engage with content multiple times at their convenience; and (2) weekly live virtual coaching sessions with an NBC-HWC focused on implementing evidence-based behavior change strategies to support patients’ chosen goals. The use of NBC-HWCs rather than licensed therapists was intentional to support scalability, expand access to care, and align the live treatment component with a structured behavior change model that complements the asynchronous CBT content.

This clinical program is meant to serve as an intermediary treatment option, balancing the benefits of both self-guided, smartphone-based CBT programs and traditional one-on-one care offered by clinicians (Table 1). Traditional psychotherapy is often constrained by provider availability and extended wait times [38,39], whereas self-guided CBT offers immediate access but may be limited by engagement and adherence challenges [40], as well as rarely, if ever, meeting WCAG standards [41]. Hybrid models that combine digital content with human support can address these limitations by maintaining scalability while incorporating structured support, which has been associated with improved engagement and more efficient use of clinical resources [42].

**Table 1.** Comparison of cognitive behavioral therapy (CBT) delivery models and access characteristics.

Feature	CBT delivery model		
	Self-guided CBT applications	Traditional provider care	Rauha CBT model
Clinician involvement	Little to none	Direct	Psychologist-developed and guided
Delivery format	Asynchronous	Synchronous	Combined
Wait times	Immediate	48-day average	3-day average
Scalability	High	Low	High
Consistency of care	Standardized content	Clinician-dependent	Standardized content with personalized coaching
Meets WCAG^a^ standards	Rare	Not applicable	Yes
Flexible to patient needs	Limited	High	High
Clinical standards	Variable	High	High
Cost efficiency^b^	Low cost, variable engagement	High cost, resource-intensive	Moderate cost, scalable support

a WCAG: Web Content Accessibility Guidelines.

b Cost efficiency reflects relative resource utilization and scalability rather than absolute cost.

### Study Objectives

The central aim of this study was to evaluate Rauha in the treatment of moderate-to-severe symptoms of anxiety and depression in adults with a hearing and/or mobility-related disability. We hypothesized that there would be (1) at least 80% treatment retention among those who initiated the intervention and (2) a statistically and clinically significant reduction (CSR) in anxiety and depressive symptoms between baseline and the end of the treatment period through a 4-week follow-up. As an exploratory aim, we assessed user acceptability, experience, and usability of the Rauha program.

## Methods

### Study Design

This study used a single-arm, within-participants design to measure the clinical impact of Rauha on symptoms of anxiety and depression, as measured by the Hamilton Anxiety Rating Scale (HAM-A) [43] and Hamilton Depression Rating Scale (HAM-D) [44], respectively. Patients were assessed with the HAM-A and HAM-D at baseline (preintervention), immediately after completing treatment (post intervention), and 4 weeks after completing treatment (follow-up). Participation from baseline to follow-up lasted approximately 12 weeks.

### Participants

Participants were recruited through approved study flyers at Endeavors Programs in San Antonio, Texas, as well as through other approved recruitment channels, including clinical and community referrals. Inclusion criteria for participation were a self-reported mobility or hearing disability, age 22 years or older, a minimum score of 10 on the Patient Health Questionnaire-9 (PHQ-9) [45] or Generalized Anxiety Disorder-7 (GAD-7) [46], access to a stable internet connection via Wi-Fi or mobile data, access to an iPhone or iPad (optionally, an iPhone could be provided by the research team), English fluency, resident of the United States for the duration of the study, no changes in psychotropic medication or psychotherapy treatment in the 30 days before study entry, and comprehension of and willingness to sign informed consent and complete all study procedures.

Exclusion criteria were a diagnosis of psychotic or bipolar disorder, active participation in another treatment trial, nontobacco substance use disorder within the past 12 months, any suicidality beyond passive ideation in the past 12 months, and currently pregnant or planning to become pregnant during the treatment period.

### Ethical Considerations

This study was approved by the Advarra Institutional Review Board (Protocol 79652). All participants provided informed consent prior to participation in any study-related activities. Participation was voluntary, and participants were informed that they could withdraw from the study at any time without penalty. Participant privacy and confidentiality were protected throughout the study. All study data were collected, transmitted, and stored within a HIPAA-compliant digital platform and secure data management dashboard with restricted access limited to authorized study personnel. Appropriate administrative, technical, and physical safeguards were implemented to protect participant information. Data were deidentified prior to analysis and reporting. Participants received US $125 in compensation for their participation in the study in accordance with the institutional review board–approved protocol and were not contingent upon study outcomes.

### Materials

#### Patient Health Questionnaire-9

The PHQ-9 is a 9-item self-report measure corresponding to the *Diagnostic and Statistical Manual of Mental Disorders, Fifth Edition* (DSM-5) [47] criteria for major depressive disorder and is used as an inclusion criterion in this study [45]. Each item is rated on a 4-point Likert scale ranging from 0 (“not at all”) to 3 (“nearly every day”), yielding total scores ranging from 0 to 27, where higher scores reflect more severe levels of depressive symptoms. The PHQ-9 has established cutoff scores of 5, 10, 15, and 20, representing mild, moderate, moderately severe, and severe depression, respectively. It has demonstrated good internal consistency (α=.85) [45].

#### Generalized Anxiety Disorder-7

The GAD-7 is a 7-item self-­report measure of anxiety symptoms corresponding to the DSM-5 [47] criteria for GAD, used as an inclusion criterion in this study [46]. Each item is rated on a 4-point Likert scale ranging from 0 (“not at all”) to 3 (“nearly every day”), yielding total scores ranging from 0 to 21, with higher scores indicating greater anxiety symptoms. The GAD-7 has established cutoff scores of 5, 10, and 15, representing mild, moderate, and severe anxiety, respectively. It has demonstrated good internal consistency (α=.89) [48].

#### Columbia Suicide Severity Rating Scale

The Columbia Suicide Severity Rating Scale (C-SSRS) is a 6-item suicide risk screening tool that assesses suicidal ideation and behavior and is used as an exclusionary criterion in this study [49]. It was administered in an interview format, first at baseline and then at the start of each coaching session. The baseline C-SSRS assessed past-month suicidality (with the exception of item 6), whereas the subsequent weekly C-SSRS was used to assess any suicidality between each coaching session. The C-SSRS has demonstrated excellent internal consistency (α=.94) [49].

#### Hamilton Anxiety Rating Scale

The HAM-A is a structured interview for examiners to rate anxiety severity, and it was used as a primary outcome measure in this study [43]. It assesses anxiety symptoms based on 14 items, each rated from 0 (“not present”) to 4 (“severe”), with scores ranging from 0 to 56. It uses established cutoff scores, where a score of 7 or lower indicates no or minimal anxiety, 8 indicates mild anxiety, 15 indicates moderate anxiety, and 24 or higher indicates severe anxiety [50]. The HAM-A has demonstrated good internal consistency (α=.89) [51].

#### Hamilton Depression Rating Scale

The HAM-D is a structured interview for examiners to rate depression severity, and it was used as an outcome measure in this study [44]. It assesses depression symptoms based on 17 items, which range from 0 (“not present”) to 4 (“severe”) with total scores ranging from 0 to 54. Among these 17 items, 8 items are rated on a 0 to 2 scale and 9 items are rated on a 0 to 4 scale. In terms of established cutoff scores, a score of 7 or lower suggests no depression, 8 indicates mild depression, 17 indicates moderate depression, and 24 or greater indicates severe depression [52]. The HAM-D has demonstrated good internal consistency (α=.81–.89) [53].

#### Participant-Reported Surveys

Three exploratory surveys comprising 35 questions were created to assess the acceptability, usability, and user experience of the Rauha DTx.

#### User Acceptability Survey

This 10-item scale measured overall satisfaction, perceived value, and personal relevance of the Rauha DTx, including how useful and worthwhile participants found its content and structure. Items were rated on a 5-point Likert scale ranging from 1 (“strongly disagree”) to 5 (“strongly agree”). Example items include “My participation on this app was worth the time” and “I am satisfied with the help I received on the app.”

#### System Usability Survey

This 10-item scale measured users’ perceptions of ease of use, efficiency, and learnability of the Rauha DTx, evaluating whether participants found the interface intuitive and consistent. Items were rated on a 5-point Likert scale ranging from 1 (“strongly disagree”) to 5 (“strongly agree”). Example items include “I think that I would like to use this system frequently” and “I feel very confident using this app.”

#### User Experience Survey

This 15-item scale measured qualitative aspects of user experience within the Rauha program, including the perceived helpfulness of program features, engagement with multimedia content, and support from mental health coaches. It captured both functional and interpersonal satisfaction with the DTx and coaching components. Items were rated on a 5-point Likert scale ranging from 1 (“strongly disagree”) to 5 (“strongly agree”). Example items include “My mental health coach was critical to my completion of the program” and “I found the mental health coaches to be a useful part of the program.”

### Procedures

#### Recruitment and Screening

Adults interested in participating in the study first initiated contact with study personnel at Endeavors Programs. All interested adults then preconsented to an initial screening with the GAD-7 and PHQ-9 to establish appropriateness for study inclusion based on anxiety and/or depressive symptoms, respectively, and a self-reported disability. Those who met these initial eligibility criteria were then invited to the study consent process, where the full study details were outlined, including verification of remaining inclusion and exclusion criteria. After signing the consent form, participants were scheduled to complete the baseline HAM-A and HAM-D. Importantly, the HAM-A and HAM-D were administered by licensed clinicians who received the same training to standardize assessment procedures and scoring to maximize interrater reliability. These clinicians were independent from the intervention team, and each participant was evaluated by the same clinician at baseline, post treatment, and follow-up to ensure consistency in assessment and minimize variability due to differences in raters across time points. Upon completion of the structured interviews, participants were invited to download the Rauha smartphone DTx, assigned a coach, and booked for their virtual coaching orientation appointment. This orientation appointment was not part of the treatment but rather was used as an introduction to assist with registering the participant on the Rauha DTx and to help prepare the participant for the treatment itself. Registration for Rauha included a brief disability and demographic screening, a program introduction video by a licensed psychologist, and a coach-led onboarding session to orient participants to the program.

#### Treatment

The Rauha program targeted both anxiety and depressive symptoms using an 8-week intervention, combining the smartphone-based, self-guided component with weekly sessions led by an NBC-HWC and implementing evidence-based behavior change strategies. The selection of an 8-week intervention period was informed by prior work on both traditional CBT and digitally delivered CBT, as well as practical considerations related to the structure of the DTx and coaching model. CBT is typically delivered as a structured, time-limited intervention, with standard protocols often spanning on the order of 6 to 12 weeks depending on the target condition and setting [54,55]. Internet-based and guided CBT programs follow a similar pattern, with many interventions delivered within a defined multiweek window and a substantial proportion clustering around approximately 7 to 9 weeks [56,57]. An 8-week duration was therefore chosen as a balance between providing sufficient time for skill acquisition and consolidation while maintaining a format that is feasible and consistent with established CBT and digital intervention models.

Each week, the CBT-based smartphone program focused on a new, overarching topic comprising multiple lessons that fulfill that topic (Multimedia Appendix 1). These lessons consist of psychoeducational videos featuring a licensed psychologist, animated videos illustrating therapeutic concepts from an example patient perspective, infographics, and brief reading lessons to teach psychoeducation and various CBT concepts, as well as interactive quizzes and exercises that allow participants to apply the skills being taught. Each weekly digital topic is designed to take approximately 60 minutes to complete and can be divided into smaller segments, allowing participants to engage with the material according to their schedule and preferences.

In addition to the 60 minutes spent in the smartphone program, participants also met with an assigned NBC-HWC for 30 minutes each week. In accordance with board certification training, coaches used a client-centered approach to support participants in identifying mental health–related goals, using behavior change strategies consistent with motivational interviewing, behavioral activation, values identification, and solution-focused behavioral therapy. At the start of each coaching session, coaches administered the C-SSRS to assess suicidality and ensure participant safety. A safety escalation protocol was put in place in the event a participant endorsed any item on the C-SSRS beyond item 2, which would entail a same-day warm transfer back to their referring clinician for further risk management and appropriate treatment planning in the event of nonimminent suicidal concerns. In the case of imminent suicidal intent, emergency services would be activated, followed by notifying the original referring provider for follow-up once immediate safety was confirmed. Following the initial safety assessment, each session proceeded according to a semistructured approach that entailed (1) a brief mood check-in, (2) review of digital CBT content according to that week, (3) reviewing and reinforcing past skills (eg, behavioral activation, values clarification), (4) setting goals for the upcoming week, and then (5) identifying and troubleshooting any barriers to continued skills practice or weekly goals. While this semistructured approach was standardized across participants, coaches were permitted to tailor certain aspects of coaching according to individual needs, so long as it aligned with the framework of the CBT program. Specifically, this tailoring was restricted to the examples used to illustrate CBT concepts, pacing of skill learning based on participant comprehension or engagement, and goal refinement based on individual readiness and unique contextual barriers. NBC-HWC coaching is standardized to support engagement, goal attainment, and real-world application of CBT skills, without providing psychotherapy or modifying CBT content.

Steps were taken to ensure coaching fidelity both before participant enrollment and throughout active intervention, trained by a licensed clinician. Before participant enrollment, coaches were required to complete standardized training in the intervention protocol, undergo ongoing supervision, and adhere to rigorous quality assurance processes, ensuring consistent and reproducible delivery across all participants. This systematic approach was used in order to provide evidence-based support with high fidelity, minimizing between-participants variability and maximizing confidence in outcomes.

Upon completion of the intervention, anxiety and depressive symptoms were assessed using the clinician-administered HAM-A and HAM-D, respectively. These assessments were then administered at 4 weeks post intervention (ie, follow-up) to evaluate the durability of symptom changes. Importantly, even after the 8-week coaching component ended, participants retained access to the smartphone-based CBT program through the 4-week follow-up period, allowing them to revisit any clinical content or exercises.

### Statistical Approach

#### Clinical Outcome Analysis

Regarding the first hypothesis, the retention rate was calculated as the proportion of participants who completed the full 8-week intervention to include all smartphone-based CBT modules and all 8 coaching sessions. All analyses related to the second hypothesis used the sample of participants who initiated treatment. Clinical measures are summarized with means and SDs for continuous measures and percentages for categorical and binary measures. Mixed-effects modeling (MEM) [58] was used for analyses focusing on symptom change. MEM accommodates the within-participant correlation over time for repeated measures, which was fit through a compound symmetry design for the variance-covariance matrix. MEM does not require balanced data per person, where missed assessments are excluded from the analysis. Time was measured discretely by assessment point corresponding to baseline, post treatment, and follow-up and treated as a fixed effect in the MEM. Linear contrasts of the model parameterization allowed us to estimate change from baseline to post treatment corresponding to the intervention effect, as well as change from baseline through follow-up, which allowed us to quantify the durability of the intervention effect. We used partial eta as an indication of within-person change. Following Cohen conventions [59], partial eta squared values of 0.1, 0.3, and 0.5 represent small, medium, and large effect sizes, respectively. Jacobson and Truax [60] criteria informed CSR calculations. Percentages with 95% CIs using the Clopper-Pearson exact CI formulas were used to calculate the proportion of participants achieving a CSR. Additionally, we calculated percent change in symptoms ranging from 10% to 90% for both anxiety and depression with 95% CIs.

#### Scoring and Analysis of Participant-Reported Surveys

Responses to the user acceptability, usability, and experience surveys were summarized descriptively at both the item and scale levels. For each item, we calculated the proportion of participants selecting each Likert-scale response option, with primary emphasis on the percentage endorsing “agree” or “strongly agree” as an indicator of a positive response. At the scale level, we computed for each participant the percentage of items endorsed as “agree” or “strongly agree,” and then averaged these values across participants to generate overall acceptability, usability, and user experience scores (reported as percentages with SDs). This approach was used given the exploratory nature of the measures and small sample size, prioritizing depth of user perspectives.

#### Replay Rate

We examined the replay rate as an exploratory indicator of engagement using platform analytics. The system passively tracks user interactions at the lesson level, including instances where participants returned to previously completed smartphone-based CBT content. A replay event was defined as any access of a completed lesson beyond the initial viewing. For each participant, replay events were summed across the 8-week intervention, and the average replay rate was calculated across participants.

#### Power Analysis

Prior to the start of the study, preplanned power calculations were based on a 2-tailed test with an α level of .05. Using a subgroup of 72 participants with disabilities reported in previous work [61], baseline HAM-D and HAM-A SDs were 9.1 and 5.5, respectively. Using these SDs with our treatment sample, there was 82.9% and 87.1% power to detect reductions of 8 and 8.5 points, respectively, on the HAM-D. Similarly, there was 78.8% and 85.6% power to detect reductions of 4.5 and 5, respectively, on the HAM-A. As such, the proposed data analysis is proof-of-concept with the goal to serve as initial evidence for a future larger study. With the exploratory nature of our investigation, corrections for multiple comparisons are generally not needed [62]. As such, the level of significance was set at .05 for all analyses.

## Results

### Study Flow and Retention

**Figure 1.**
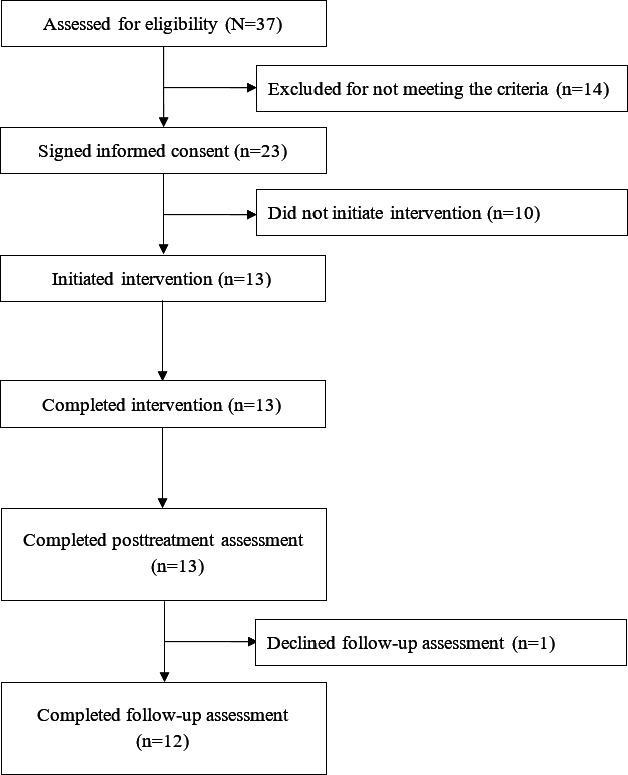
CONSORT (Consolidated Standards of Reporting Trials) flow diagram of participant progress through the study.

A total of 37 individuals were assessed for eligibility, of whom 14 were excluded for not meeting inclusion criteria. Twenty-three participants provided informed consent. Of these, 10 did not initiate the intervention: 1 declined to participate and did not complete baseline HAM-A or HAM-D assessments, and 9 elected not to continue due to preference for live clinician therapy, time-commitment requirements, discomfort with recorded sessions, or inability or unwillingness to complete the intervention on an iOS device. The remaining 13 participants initiated and completed the intervention, with no attrition during treatment, and all 13 completed the post treatment assessment. At follow-up, 12 participants completed the follow-up assessment, with 1 participant declining follow-up participation. Participant flow of the study is shown in Figure 1 (CONSORT [Consolidated Standards of Reporting Trials] diagram), detailing screening, enrollment, intervention initiation and completion, post treatment assessment, and follow-up assessment.

The final analytic sample consisted of 13 adults, of whom 53.8% (n=7) were female participants and 46.2% (n=6) were male participants. Reporting of age and race or ethnicity was optional; 12 of 13 participants reported age and 9 of 13 reported race or ethnicity. Among those reporting age, the mean age was 34.42 (SD 13.51) years. Of the 9 participants reporting race or ethnicity, 5 identified as White, 2 as two or more races, 1 as Hispanic, and 1 as Black or of African descent. With respect to disability status, 6 participants reported a mobility-related disability, 2 reported a hearing-related disability, and 5 reported both mobility-related and hearing-related disabilities. Based on baseline HAM-A and HAM-D scores, 10 participants exhibited clinically elevated levels of both anxiety and depression, 2 exhibited elevated depression only, and 1 participant did not demonstrate clinically elevated anxiety or depression despite meeting inclusion criteria. No participants endorsed any suicidal symptoms.

### Primary Outcomes

Table 2 provides descriptive statistics for HAM-D and HAM-A at our 3 assessment points.

**Table 2.** Clinical descriptives by assessment time point (N=13).

Assessment	HAM-D^a^, mean (SD)	HAM-A^b^, mean (SD)
Baseline	19.92 (5.14)	21.69 (9.35)
Post treatment	7.54 (6.63)	9.85 (8.9)
Follow-up	7.17 (5.36)	9 (7.01)

a HAM-D: Hamilton Depression Rating Scale.

b HAM-A: Hamilton Anxiety Rating Scale.

**Figure 2.**
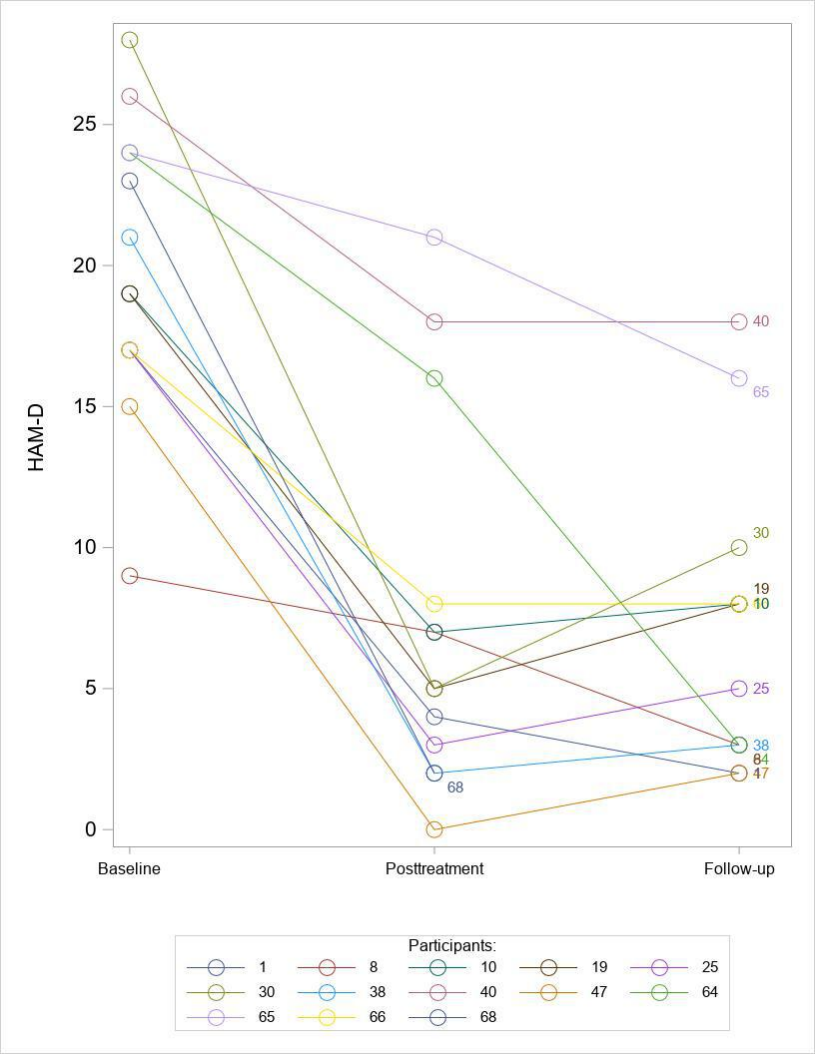
Individual profiles measured using the Hamilton Depression Rating Scale (HAM-D).

**Figure 3.**
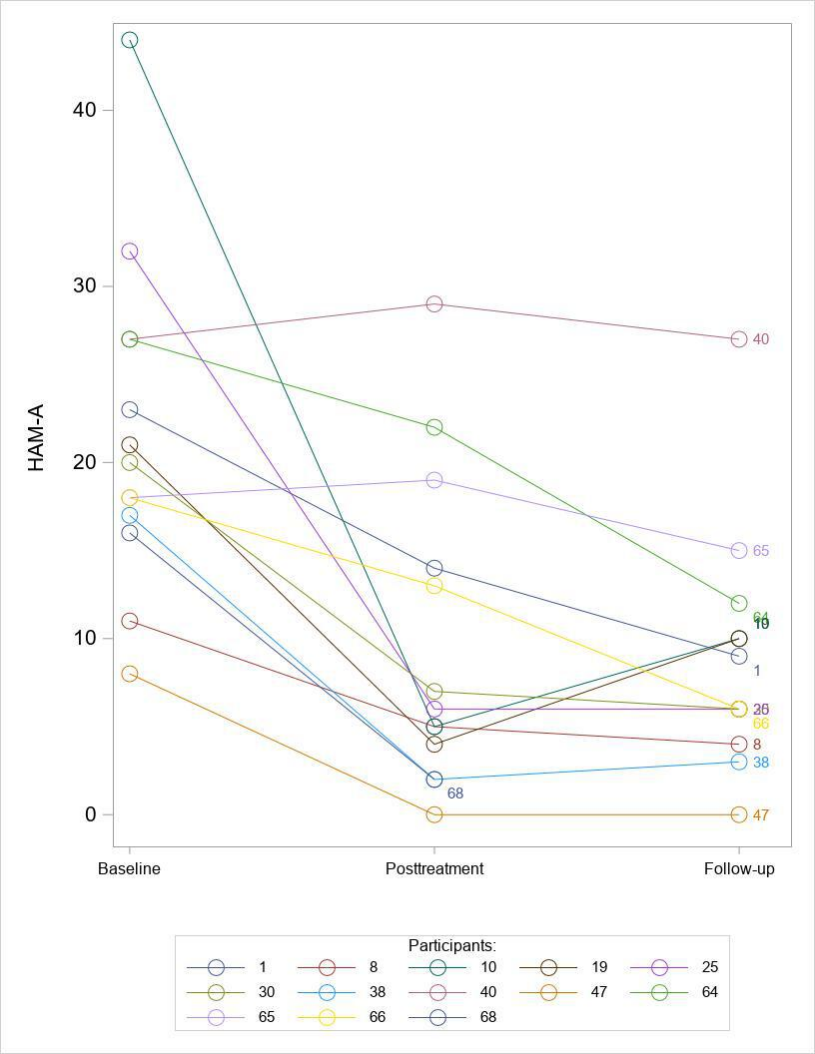
Individual profiles measured using the Hamilton Anxiety Rating Scale (HAM-A).

**Figure 4.**
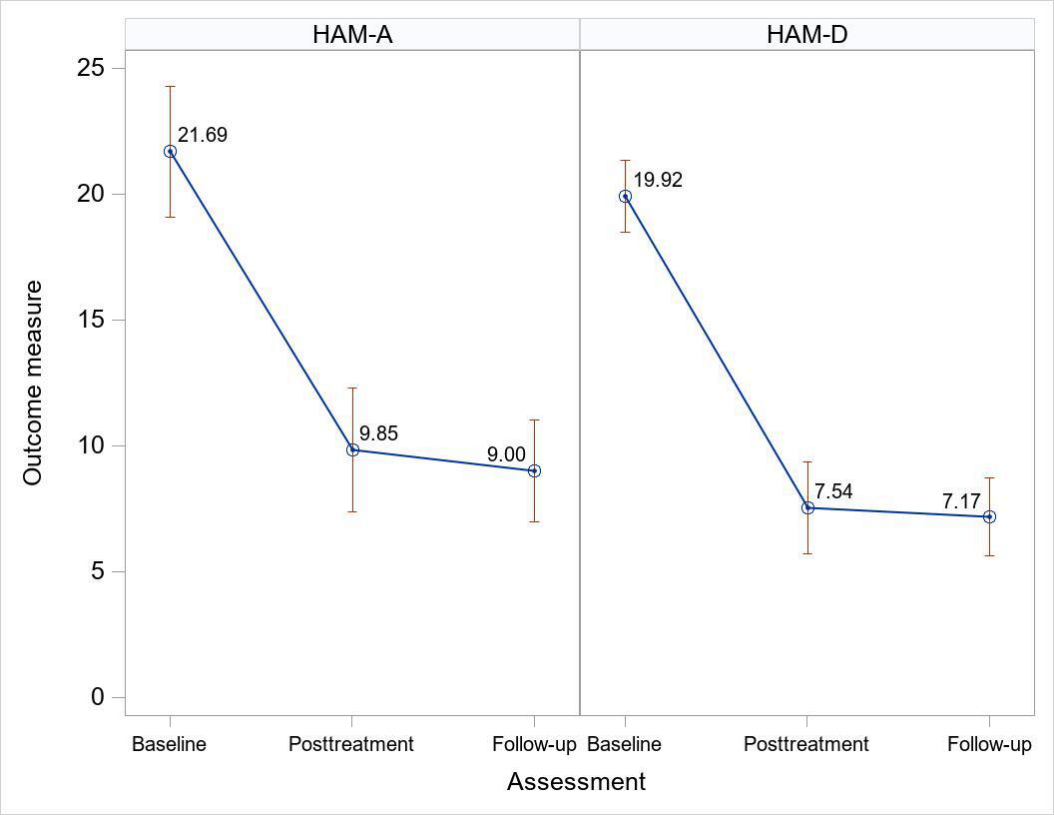
Mean profile for Hamilton Anxiety (HAM-A) and Hamilton Depression (HAM-D).

Individual spaghetti plots are illustrated in Figure 2 for HAM-D and in Figure 3 for HAM-A. Both figures illustrate 2 major findings in our study: (1) the majority of participants improved over the observational period and (2) the completeness of the data, where all participants except 1 (ID=68, who did not provide follow-up assessment) completed all 3 assessments. Figure 4 illustrates the mean profile over time for both anxiety (HAM-A) and depression (HAM-D).

With respect to anxiety, we observed a significant average reduction of 11.84 (SE 2.44) from baseline (mean 21.69, SD 9.35) to post treatment (mean 9.85, SD 8.90; *t*_13_=4.85, *P*<.001). From post treatment (mean 9.85, SD 8.90) to follow-up (mean 9.00, SD 7.01), we observed a nonsignificant reduction of 1.24 (SE 2.51; *t*_13_=0.48, *P*=.64). Cumulatively from baseline (mean 21.69, SD 9.35) to follow-up (mean 9.00, SD 7.01) for anxiety, we observed a significant average reduction of 13.05 (SE 2.51; *t*_13_=5.21, *P*<.001), corresponding to a mean shift from moderate to mild anxiety between baseline and follow-up. Partial η estimates quantifying the size of the effect for the change from baseline to post treatment and follow-up were η²_p_=0.80 (95% CI 0.48-0.90) and η²_p_=0.82 (95% CI 0.52-0.91), respectively, corresponding to large effects. Similarly, for depression, we observed a significant average reduction of 12.39 (SE 1.50) from baseline (mean 19.92, SD 5.14) to post treatment (mean 7.54, SD 6.63; *t*_13_=8.24, *P*<.001). From post treatment (mean 7.54, SD 6.63) to follow-up (mean 7.17, SD 5.36), we observed a nonsignificant reduction of 0.44 (SE 1.55; *t*_13_=0.29, *P*=.78). Cumulatively from baseline (mean 19.92, SD 5.14) to follow-up (mean 7.17, SD 5.36), we observed a significant average reduction of 12.83 (SE 1.55; *t*_13_=8.30, *P*<.001), corresponding to a mean shift from moderate to mild/nondepressed between baseline and follow-up. Partial η estimates quantifying the size of the effect for the change from baseline to post treatment and follow-up were η²_p_=0.92 (95% CI 0.76-0.95) and η²_p_*=*0.92 (95% CI 0.76-0.96), respectively, corresponding to large effects.

Based on published research, we define CSR as a 4-unit change in both HAM-A [63] and HAM-D [64]. Using all 13 participants, with nonresponse corresponding to not achieving CSR, at post treatment, 84.6% (95% CI 54.5%-98.0%) achieved CSRs for both anxiety and depression. At follow-up, 76.9% (95% CI 46.2%-95.0%) achieved a CSR in anxiety and 92.3% (95% CI 74.0%-99.0%) in depression. Interestingly, 1 participant (ID=8) started the study without an elevation in the HAM-A or HAM-D but still displayed a CSR in both. As CSR can depend on initial measurement score, the percentage of participants achieving a symptom reduction ranging from 10% to 90% (in 10% increments) from baseline to post treatment and baseline to follow-up are shown in Tables3  4, respectively.

**Table 3.** Percentage of participants reaching percentage thresholds of symptom reduction from baseline to post treatment (N=13).^a^

Symptom reduction^a^	HAM-D^b^, percentage of participants (95% CI^c^)	HAM-A^d^, percentage of participants (95% CI^c^)
10% reduction	100 (75.3‐100)	84.6 (54.6‐98.1)
20% reduction	92.3 (64.0‐99.8)	76.9 (46.2‐95.0)
30% reduction	84.6 (54.6‐98.1)	69.2 (38.6‐90.9)
40% reduction	69.2 (38.6‐90.9)	61.5 (31.6‐86.1)
50% reduction	69.2 (38.6‐90.9)	61.5 (31.6‐86.1)
60% reduction	61.5 (31.6‐86.1)	53.8 (25.1-80.8)
70% reduction	53.8 (25.1‐80.8)	46.2 (19.2-74.9)
80% reduction	38.5 (13.9‐68.4)	46.2 (19.2-74.9)
90% reduction	23.1 (5.04‐53.8)	7.69 (0.19-36.0)

a Thresholds of symptom reduction from baseline to post treatment in 10% increments.

b HAM-D: Hamilton Depression Rating Scale.

c 95% CIs are Clopper-Pearson exact confidence bounds.

d HAM-A: Hamilton Anxiety Rating Scale.

**Table 4.** Percentage of participants reaching percentage thresholds of symptom reduction from baseline to follow-up (N=12).^a^

Symptom reduction^b^	HAM-D^c^, percentage of participants (95% CI)	HAM-A^d^, percentage of participants (95% CI)
10% reduction	100 (75.3-100)	91.70 (61.5-99.8)
20% reduction	100 (75.3-100)	83.30 (51.6-97.9)
30% reduction	100 (75.3-100)	83.30 (51.6-97.9)
40% reduction	83.30 (51.6-97.9)	83.30 (51.6-97.9)
50% reduction	83.30 (51.6-97.9)	83.30 (51.6-97.9)
60% reduction	58.30 (27.7-84.8)	66.70 (34.9-90.1)
70% reduction	41.70 (15.2-72.3)	41.70 (15.2-72.3)
80% reduction	33.30 (9.92-65.1)	25.00 (5.49-57.2)
90% reduction	0.00 (0.00-26.5)	8.33 (0.21-38.5)

a One participant was excluded due to not providing follow-up data. 95% CIs are Clopper-Pearson exact confidence bounds.

b Thresholds of symptom reduction from baseline to 4-week follow-up in 10% increments.

c HAM-D: Hamilton Depression Rating Scale.

d HAM-A: Hamilton Anxiety Rating scale.

**Figure 5.**
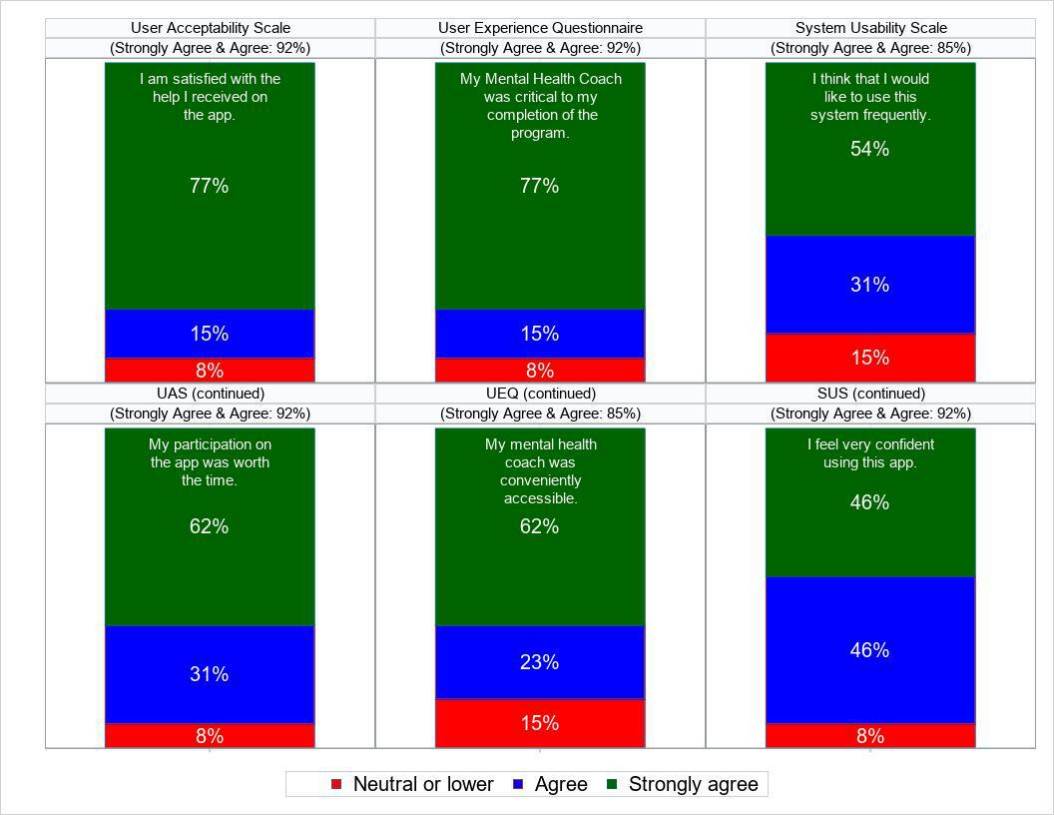
Ratings of acceptability, user experience, and usability of the Rauha program. UAS: User Acceptability Scale; UEQ: User Experience Questionnaire; SUS: System Usability Scale.

Figure 5 summarizes the acceptability, experience, and usability of the Rauha smartphone platform. Among participants who agreed or strongly agreed based on direct DTx experience, 85.6% (SD 10.4%) reported positive acceptability, 87.7% (SD 8.3%) reported positive experience, and 85.0% (SD 6.3%) reported positive usability. The most highly endorsed items (>90%) highlight the aspects of the program driving the positive user experience: overall satisfaction with the treatment program, relevance of treatment strategies to daily life, the central role of mental health coaches in treatment success, use of video and text delivery within Rauha, ease of understanding digital content, and confidence in using the platform. Importantly, there was no negative feedback on any item across these self-report surveys and no accessibility issues, given the WCAG 2.2 AA standards of the smartphone program. These findings underscore that participants valued not just general satisfaction but specific program components that supported engagement, skill application, and confidence in using Rauha effectively.

Regarding replay rate, participants revisited previously completed CBT lessons multiple times over the course of the intervention, with an average replay rate of 5.5. Replay behavior was derived from platform usage analytics, with some modules showing higher repeat access based on user interaction patterns (eg, repeated entry and time spent within lessons). In the context of consistently high acceptability, experience, and usability ratings, this pattern of re-engagement is more consistent with voluntary reuse of content than with difficulty understanding the material.

## Discussion

### Principal Findings

The central aim of this study was to evaluate Rauha—a mental health DTx intervention combining an asynchronous, CBT-based treatment with a synchronous NBC-HWC implementing behavior change strategies—in treating symptoms of moderate-to-severe anxiety and depression in adults with hearing and/or mobility-related disabilities. We hypothesized that (1) there would be a treatment retention rate of at least 80%, and (2) there would be a CSR in internalizing symptoms from pretreatment to post treatment and through follow-up. The data supported both hypotheses, with a retention rate of 100% and a statistically and clinically significant decrease in both anxiety and depression symptoms on average that was sustained during the 4-week follow-up. Furthermore, this average decrease in symptoms far exceeded the minimum threshold for clinical significance by a magnitude of 3, such that participants had even more benefit than expected.

This is the first known study to assess the treatment effect of a WCAG 2.2 AA–certified platform-aligned DTx combined with a weekly, virtual NBC-HWC to treat moderate to severe symptoms of anxiety and depression in adults with disabilities. In addition, as noted previously, 1 participant with subclinical baseline levels of both anxiety and depression nevertheless achieved a CSR despite a generally lower capacity for symptom change among those with lower baseline severity [9]. Given these results, this study offers strong preliminary data to support this novel approach to treatment. In addition, the 100% retention rate also speaks to the acceptability and engagement of the treatment itself, given that treatment retention is often an issue both in research [65] and clinical [66] settings. These findings extend the overall evidence of CBT-based interventions to less-used treatment modalities while also being applied to an underserved clinical population, adults with disabilities and functional limitations. The clinically significant outcomes from this study are either commensurate with or exceed many previous outcome studies, including internet-based CBT [18,67,68], traditional CBT delivered as weekly individual therapy [69-71], combined internet-based and individual face-to-face CBT [70,72-74], and CBT combined with pharmacotherapy [75].

At a systems level, and as it relates to barriers prohibiting access to adequate treatment, both of the Rauha treatment components can increase overall access to care, as self-guided treatment does not require time from a provider for a patient to receive care, and there are over 12,000 NBC-HWCs that can be considered part of the otherwise limited mental health resources to drastically expand treatment options [76]. Thus, CBT and other evidence-based approaches can be highly scaled with this approach without sacrificing clinical impact.

### Strengths and Limitations

This study has several notable strengths. First, it evaluated a novel, digitally delivered CBT intervention targeting an underserved population with nuanced physical and mental health needs, addressing a critical gap in accessible mental health care. These findings extend our understanding of the potential for nontraditional treatment modalities and suggest that CBT can remain clinically impactful even when delivered through a smartphone-based DTx. Second, the use of clinical significance, in addition to statistical significance, provides a richer assessment of treatment impact, highlighting the clinical meaningfulness of Rauha for patients. Third, the inclusion of a 4-week postintervention follow-up allowed for a preliminary assessment of the durability of symptom improvements. While this time frame provides early support for the maintenance of treatment effects, longer follow-up periods are needed to fully characterize sustained outcomes in anxiety and depression. Interestingly, there was an increase in clinically significant improvement in depression between post treatment and follow-up, which may be attributable to participants having continued access to the smartphone-based CBT program to revisit relevant clinical content, though the robustness of such a finding would require further research. Finally, assessing user acceptability, experience, and usability offers greater insight into the participant’s experience with the DTx, demonstrating highly positive views regarding the ease of use, DTx content, and coaching sessions. Together, these elements underscore the potential of Rauha as a scalable, accessible, and evidence-informed treatment.

This study has several limitations that should be considered when interpreting the findings. First, the sample size was small, which limits statistical power, the precision of estimates, and the generalizability of findings. Accordingly, the observed large effect sizes should be interpreted with some caution and considered preliminary support for proof-of-concept requiring replication in higher-powered clinical studies. Second, the participant population was limited to individuals with hearing and mobility disabilities, and researchers allowed certain demographic disclosures to be optional for participant comfort. Future efforts could expand representation across a broader range of functional and accessibility challenges and require demographic reporting, which would enhance generalizability. Third, the study did not include a control condition, which limits formal causal inference such that results may reflect uncontrolled factors (eg, natural symptom fluctuation, placebo effects, regression to the mean, or untracked changes in medication during the intervention). Given this, future studies incorporating a control condition are planned to rigorously assess causal treatment effects. Fourth, participants were recruited in part through a mental health clinic, which may introduce selection bias, as the individuals referred may differ from the broader population in terms of treatment readiness, clinical profiles, and motivation to engage in care. Future studies can address this by using more diverse recruitment strategies to strengthen generalizability. Finally, because Rauha combines a self-guided digital component with NBC-HWC support, it is not possible to attribute the observed outcomes to either component alone. That is, the present design does not allow for attribution of effects to either the digital CBT component or the coaching component independently. Future studies using component analyses or factorial designs are needed to isolate the mechanisms of change and optimize the intervention itself by identifying the most impactful factors. Collectively, these limitations highlight the need for continued research while underscoring the promising potential of Rauha to improve outcomes in this population.

### Conclusions

To our knowledge, this is the first study to evaluate a hybrid digital CBT intervention—combining self-guided, smartphone-delivered CBT with live weekly sessions led by a NBC-HWC—for adults with disabilities experiencing anxiety and/or depression. The findings provide preliminary support for clinical benefit of Rauha, high engagement, accessibility, and scalability, while identifying important directions for future research. Importantly, this work supports the potential of a coordinated digital CBT and coaching model to address persistent barriers to care and deliver flexible, clinically meaningful support to populations historically underserved in mental health services. Overall, Rauha represents a promising, evidence-informed approach that extends the reach of CBT and highlights the value of integrating DTx with coaching to improve mental health outcomes among adults with functional and accessibility challenges.

## Supplementary material

10.2196/92448 Multimedia Appendix 1 Rauha 8-week cognitive behavioral therapy digital therapeutic program.

## Abbreviations

C-SSRS: Columbia Suicide Severity Rating Scale
CBT: cognitive behavioral therapy
CONSORT: Consolidated Standards of Reporting Trials
CSR: clinically significant reduction
DSM-5: Diagnostic and Statistical Manual of Mental Disorders, Fifth Edition
DTx: digital therapeutic
GAD-7: Generalized Anxiety Disorder-7
HAM-A: Hamilton Anxiety Rating Scale
HAM-D: Hamilton Depression Rating Scale
HIPAA: Health Insurance Portability and Accountability Act
MEM: mixed-effects modeling
NBC-HWC: National Board-Certified Health and Wellness Coach
PHQ-9: Patient Health Questionnaire-9
WCAG: Web Content Accessibility Guidelines
