# Supplementary material for: Evaluating a Dual Digital Cognitive Behavioral Therapy and Health and Wellness Coaching Intervention for Anxiety and Depression: Single-Arm Pilot Study
Source: JMIR Hum Factors. 2026 Jun 25;13:e92448. doi: 10.2196/92448 (PMC13295422; doi:10.2196/92448)
Supplement: Multimedia Appendix 1 [file humanfactors-v13-e92448-s001.docx]

**Online Supplemental Materials**

| *Rauha*® *8-Week Cognitive Behavioral Therapy Digital Therapeutic Program* | | |
| --- | --- | --- |
| Week | Topic | Lessons |
| 1 | Introduction to CBT & Goal Setting | - Introduction to Rauha® and the CBT model - Understanding emotions - Cognitive distortions - Goal Setting |
| 2 | Behavioral Activation & Sleep | - Behavioral activation - Using an activity log - Scheduling pleasant activities - Sleep hygiene & creating a sleep plan |
| 3 | Reducing Avoidance & Rumination | - Benefits of behavior change - Identifying avoidance - Rumination as avoidance |
| 4 | Introducing Automatic Thoughts & Thought Records | - Thoughts influencing emotions - Introduction to thought record - Recognizing automatic thoughts |
| 5 | Alternative Responses & Core Beliefs | - Forming alternative responses - Common obstacles to thought records - Identifying core beliefs |
| 6 | Exposure Exercises, Phobias & Social Anxiety | - Anxiety and avoidance - Exposure therapy exercises - Phobias - Social anxiety |
| 7 | Generalized Anxiety & Panic Attacks | - Exposure therapy obstacles - Generalized anxiety - Panic attacks - Anxiety management strategies |
| 8 | Relationships, Communication & Maintaining Progress | - Relationships & communication styles - Assertiveness training - Reviewing & revising goals - Treatment maintenance |
| *Note.* CBT = Cognitive Behavioral Therapy. | | |
